# The Utility of the WMS‐IV LM Subtest as an Inclusionary Measure in Early AD Trials

**DOI:** 10.1002/alz70859_107526

**Published:** 2025-12-25

**Authors:** Jessica Stenclik, Erica R. Appleman, Andrei Iacob, Amanda Hackebeil, Sayaka Machizawa

**Affiliations:** ^1^ Signant Health, Blue Bell, PA USA

## Abstract

**Background:**

The Mini Mental State Examination (MMSE) is a commonly used inclusionary screening measure of cognition in Alzheimer’s disease (AD) trials. The Wechsler Memory Test‐IV Logical Memory (WMS‐IV LM) subtest has emerged as an additional screening measure to assess episodic memory loss and assist with appropriate enrollment for trials recruiting early AD participants. The WMS‐IV LM II delayed memory score has been utilized for inclusion based on score ranges indicative of episodic memory loss by age The WMS‐IV LM is more time‐intensive and complex for raters to administer. This study investigated the utility of adding WMS‐IV LM at screening.

**Method:**

Data from two multinational Phase 2 AD trials that utilized the MMSE and WMS‐IV LM as screening measures were included. eCOA alerts were programmed to trigger if a participant did not meet MMSE and/or WMS‐IV LM II inclusionary criteria. These alerts were used to explore the rate of exclusion for each measure. Correlation between WMS‐IV LM II and MMSE total scores was also examined.

**Result:**

2517 screening visits where both the MMSE and WMS‐IV LM were administered were included in the analysis. Of these 2517 screening visits, 43% (1089/2517) did not trigger either the WMS‐IV LM II or MMSE exclusion alert. The MMSE exclusion alert alone triggered in 22.73% of the visits (572/2517) and the WMS‐IV LM II exclusion alert alone triggered in 31.70% (798/2517) of the visits. MMSE total scores and WMS‐IV LM II scores were weakly correlated r(2516) = .38, *p*< .001.

**Conclusion:**

The present study found that less than half of participants did not screen fail due to MMSE or WMS‐IV LM II inclusionary criteria. About 30% of participants did not screen fail on the MMSE but proceeded to screen fail on the WMS‐IV LM II. This indicates that the WMS‐IV LM II, which is being used as an indicator of episodic memory impairment, provided utility above and beyond a more general and brief screening tool such as the MMSE in these AD trials. Although the WMS‐IV LM II may be cumbersome and lengthy to administer, it appears useful in improving classification of early AD participants.

